# 4-Phenylbutyric Acid Treatment Reduces Low-Molecular-Weight Proteinuria in a *Clcn5* Knock-in Mouse Model for Dent Disease-1

**DOI:** 10.3390/ijms25158110

**Published:** 2024-07-25

**Authors:** Ana Perdomo-Ramírez, Elena Ramos-Trujillo, Jose David Machado, Victor García-Nieto, Glorián Mura-Escorche, Félix Claverie-Martin

**Affiliations:** 1 Unidad de Investigacion, Hospital Universitario Nuestra Señora de Candelaria, Instituto de Investigacion Sanitaria de Canarias (IISC), 38010 Santa Cruz de Tenerife, Spain; aperdomr@ull.edu.es (A.P.-R.); gmuraesc@ull.edu.es (G.M.-E.); 2 Seccion Medicina, Departamento de Medicina Fisica y Farmacologia, Facultad de Ciencias de la Salud, Universidad de La Laguna, 38200 Santa Cruz de Tenerife, Spain; jdmacha@ull.edu.es; 3 Unidad de Nefrologia Pediatrica, Hospital Universitario Nuestra Señora de Candelaria, 38010 Santa Cruz de Tenerife, Spain; vgarcianieto@gmail.com

**Keywords:** Dent disease-1, knock-in mouse model, sodium 4-phenylbutyrate, low-molecular-weight proteinuria

## Abstract

Dent disease-1 (DD-1) is a rare X-linked tubular disorder characterized by low-molecular-weight proteinuria (LMWP), hypercalciuria, nephrolithiasis and nephrocalcinosis. This disease is caused by inactivating mutations in the *CLCN5* gene which encodes the voltage-gated ClC-5 chloride/proton antiporter. Currently, the treatment of DD-1 is only supportive and focused on delaying the progression of the disease. Here, we generated and characterized a *Clcn5* knock-in mouse model that carries a pathogenic *CLCN5* variant, c. 1566_1568delTGT; p.Val523del, which has been previously detected in several DD-1 unrelated patients, and presents the main clinical manifestations of DD-1 such as high levels of urinary b2-microglobulin, phosphate and calcium. Mutation p.Val523del causes partial ClC-5 retention in the endoplasmic reticulum. Additionally, we assessed the ability of sodium 4-phenylbutyrate, a small chemical chaperone, to ameliorate DD-1 symptoms in this mouse model. The proposed model would be of significant value in the investigation of the fundamental pathological processes underlying DD-1 and in the development of effective therapeutic strategies for this rare condition.

## 1. Introduction

Dent disease is a monogenic proximal tubulopathy linked to the X chromosome that is characterized by an incomplete renal Fanconi syndrome, low-molecular-weight proteinuria (LMWP), hypercalciuria, nephrocalcinosis and nephrolithiasis. Between the third and fifth decade of life, more than two-thirds of patients reach end-stage renal disease (ESRD) [1]. This disease mainly affects male children; female carriers are usually asymptomatic, although a few cases have been described in girls and young women presenting a partial or even complete phenotype due to a skewed inactivation of the X chromosome [1,2,3,4,5]. Approximately 60% of cases are caused by mutations in the *CLCN5* gene (Dent disease-1, DD-1), which encodes for the ClC-5 chlorine/proton exchanger, located mostly in early endosomes of the proximal tubule [3,6,7,8].

The mechanism by which mutations in the *CLCN5* gene induce DD-1 remains unclear. Traditionally, and given the nature of the ClC-5 protein, the most widespread hypothesis is a defect in endosome acidification, which is essential for ligand dissociation and correct sorting along the endocytic pathway, although some studies have shown the existence of pathogenic mutations that do not cause endosomal acidification damage [9,10]. In the year 2000, the first *Clcn5* knock-out (KO) mice were generated; the Jentsch and Guggino models, which exhibited the phenotypic characteristics of DD-1 [11,12]. The studies carried out in both models showed that the inactivation of ClC-5 caused a loss of the endocytosis process, both in the fluid and in the receptor-mediated phases, and a loss of the expression of megalin and cubilin in the cell membrane of proximal tubule cells, which would explain the presence of LMWP [11,12,13]. On the other hand, ClC-5 function is needed to preserve suitable endosomal pH and chloride concentration in the proximal tubule (PT). Although ClC-5 is mainly co-localized with vacuolar H^+^-ATPase in renal cells, it is not obvious how it modulates its activity [8,14]. In these KO mouse models, the expression of vacuolar H^+^-ATPase is preserved but altered expression has been reported in some DD-1 patients [11,15]. Recently, the importance of functional coupling between ClC-5 and V-ATPase for proper endosomal acidification and maturation has been confirmed [16]. Additionally, there is increased evidence that ClC-5 and other ion exchangers of the ClC family exchange one H^+^ for two Cl^−^ anions, and that ClC-5 is required to support constant import of protons by the V-ATPase [17].

In 2010, Novarino et al. generated a *Clcn5* knock-in (KI) mouse [9]. Unlike KO mice, where gene expression is null, KI mice generally express a mutated protein. These KI models are used as a complementary or alternative strategy to the KO mouse and have several applications, including the study of the physiological role of the specific disease-causing mutation in humans. In this KI model, a mutation, p.Glu211Ala, not detected in any patient, was introduced. This variant uncouples Cl^−^/H^+^ transport, thereby converting the mutant protein into a Cl^−^ channel. This KI mouse presented the same phenotype as the KO mouse. However, normal levels of endosomal acidification were observed, which implies that damage to this mechanism does not provide a sufficient explanation of what happens in DD-1 [9].

We cannot discard other mechanisms by which loss of ClC-5 might modify membrane traffic such as via effects on lipid composition or dynamics [18]. A study that compared gene expression in dissected PTs of ClC-5 KO and WT mice described that the majority of pathways considerably affected are related to lipid metabolism, and particularly to fatty acid and cholesterol metabolism [19]. Nevertheless, transcriptional changes in lipid metabolism genes have not been established in DD-1 patients. 

KO mice models have been very useful for understanding the pathogenesis of DD-1; however, as they do not express the ClC-5 protein, their use for the development of therapeutic approaches has been limited. More recently, a DD-1 KO mouse model was generated using CRISPR-Cas9 technology and used to test kidney delivery of human *CLCN5* cDNA in a lentivirus-mediated gene therapy strategy. However, the therapeutic effects initially observed disappeared after the third month and were not recovered, probably due to the activation of the immune response against the transgenic product [20].

At the moment, there is no specific therapy for DD-1 patients, only supportive measures aimed at slowing the progression of kidney disease. Eventually, patients that progress to ESRD undergo hemodialysis followed by kidney transplantation [1,2]. No drug has been developed for this rare pathology, and there are currently no pharmacological clinical trials related to DD-1 (EU Clinical Trials Register https://www.clinicaltrialsregister.eu/, NIH Clinical Center Trials https://clinicaltrials.gov/, WHO Clinical Trials Search Portal https://www.who.int/clinical-trials-registry-platform/the-ictrp-search-portal, all accessed on 20 April 2024).

Here, we present the generation and characterization of a new *Clcn5* KI mouse model carrying mutation p.Val523del, which has been detected in several unrelated patients from different countries [21,22,23,24,25], hence it may be a possible mutation hotspot of the *CLCN5* gene. This mutation causes the deletion of valine 523, located in the P helix of the ClC-5 protein that is involved in the conformation of the ClC-5 homodimer. Therefore, it is expected that this deletion may result in destabilization of the structure or the prevention of interaction between the two monomers [26,27]. On the other hand, it has been shown that mutation p.Val523del reduces ClC-5 expression both at the mRNA and protein levels [28], and electrophysiological studies of this mutation in HEK293 cells demonstrated that it resulted in the complete elimination of currents [21]. Additionally, we studied the potential effect of sodium 4-phenylbutyrate (4-PBA) in this model in order to ameliorate the effects of the disease. Various studies have shown that the use of low-molecular-weight chemical chaperones can alleviate the symptoms of several renal, cardiovascular, neurological, endocrine and respiratory diseases [29,30,31,32,33,34]. Previous assays have shown that 4-PBA treatment significantly reduced 24 h urinary albumin excretion in the urine of mice with acute kidney injury [33]. Its therapeutic potential has been established in models of different diseases in vivo and in vitro, where it has been shown that it attenuates endoplasmic reticulum stress and decreases apoptosis and pyroptosis in renal tubule cells, as well as renal interstitial fibrosis [35,36,37,38]. The lack of animal models for DD-1 that express a mutant ClC-5 protein and mimic the phenotype of this disease hinders the study of disease mechanisms and the evaluation of potential new therapies. Addressing this gap would be of great value in the investigation of DD-1. Our main objective was to develop an animal model for DD-1 that would be useful for the preclinical evaluation of molecules with therapeutic potential, such as low-molecular-weight chaperones. Our KI model complements the existing ones and provides a tool for the study of possible treatments aimed at recovering the phenotype of the ClC-5 protein.

## 2. Results

### 2.1. Generation of Clcn5 Val523del Knock-in Mice

**Figure 1:**
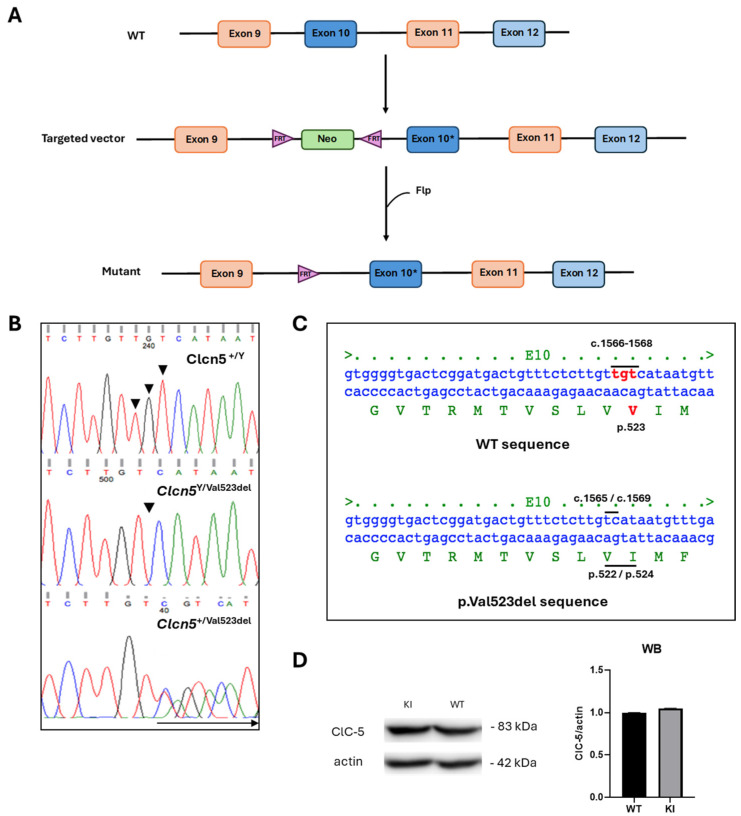
Generation of *Clcn5* Val523del knock-in. (**A**) Schematic representation of the gene targeting strategy. The diagram shows the wild-type (WT) *Clcn5* locus, targeting vector and targeted locus after Flt homologous recombination. The targeted construct contained the *Clcn5* homologous regions (exons 8 to 12 and flanking exons) with the TGT 1566–1568del mutation (*) in exon 10 and an FTR-flanked neomycin cassette (Neo) inserted in intron 9. After homologous recombination, this cassette was excised by Flt recombination. (**B**) Examples of electropherograms of direct DNA sequencing of PCR products containing the deletion site in exon 10 of WT mouse, KI and carrier female, respectively. The arrow indicates region of overlap between WT and mutant sequences (**C**) Deletion of nucleotides 1566–1568 (TGT) leads to the loss of valine 523 in the ClC-5 protein. Nucleotides in red are those affected by the deletion (**D**) Western blot of ClC-5 expression in kidney and quantification.

*Clcn5* Val523del KI mice were generated by homologous recombination in ES cells to create a mutant allele (PolyGene Transgenetics, Rümlang, Switzerland) (Figure 1A). Southern blot analysis of *Sph*I-digested DNA derived from six potentially positive ES clones resulted in a band of 12.3 Kb that confirmed the correct homologous recombination, as opposed to the band of 15.4 Kb, which corresponds to the size of the normal allele (Supplementary Figure S1). The positive clones were injected into C57BL/6 ES blastocysts, implanted in pseudo pregnant females and chimera mice were generated. Chimeras were bread to germ-line Flp-expressing mice for neomycin cassette removal. The quantification obtained by the average of two independent Western blots carried out showed that, unlike the KO models, the KI model expressed the mutant ClC-5 protein and also in the same amount as the WT, as shown in Figure 1D.

### 2.2. Genotyping

Sequencing analysis of genomic DNA of the mice generated showed that the KI mice had a deletion of the TGT nucleotides corresponding to the loss of valine 523 in the ClC-5 protein (Figure 1C). These nucleotides do appear in WT mice. In the case of females, the carriers presented a double reading from the deletion, which corresponds to the overlap of the WT and the mutated allele (Figure 1B).

### 2.3. Phenotypic Characterization of Clcn5 Val523del Knock-in Mice

**Figure 2:**
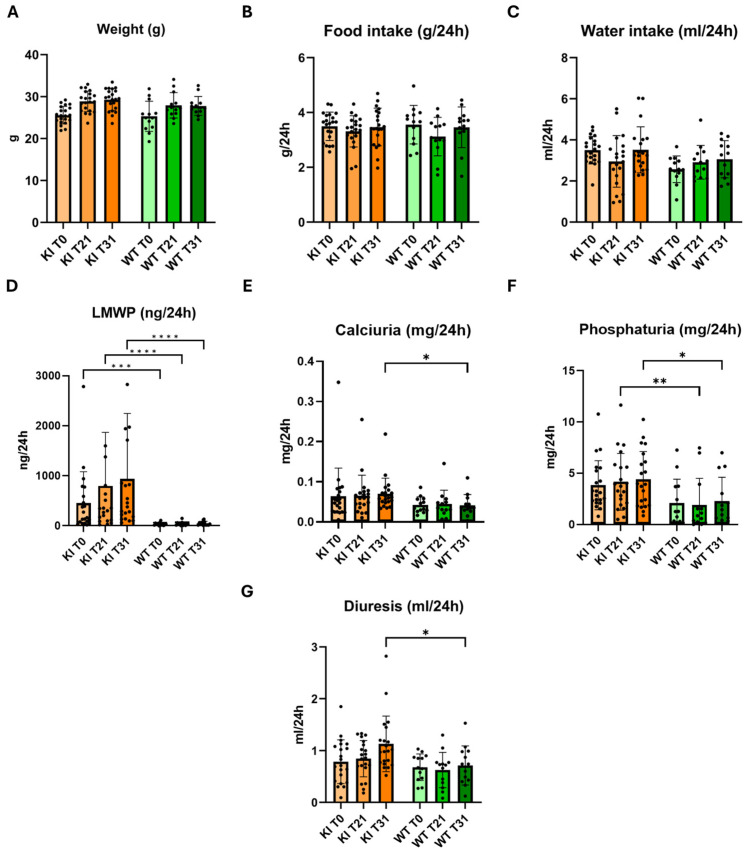
Phenotype of the *Clcn5* Val523del knock-in mouse model. (**A**–**G**) Comparison between KI and controls of weight, food and water intake, LMWP (β-2-microglobulin), calciuria, phosphaturia and diuresis. The bars correspond to the mean and standard deviations. Orange and green bars correspond to KI and control (WT) mice, respectively. *, **, *** and **** correspond to *p* < 0.05, *p* < 0.01, *p* < 0.001 and *p* < 0.0001 vs. Control.

The *Clcn5* Val523del KI mice (KI) were fertile, the number of offspring per litter was normal, as was the percentage of females and males. No survival studies were performed, since the experiment ended at four months of age, although one litter was maintained for eight months and showed no differences in aging compared to WT mice. All mice selected for characterization were males. The evolution of size was measured by weight, in which no significant changes were observed (Figure 2A). In addition, as shown by the data on water and food ingested, the KI mice did not show significant differences when it came to feeding (Figure 2B,C). Since LMWP (measured as β-2-microglobulin), increased calcium and phosphate excretion in urine and increased diuresis are some of the indicators of DD-1, these parameters were measured and compared between KI and WT mice (Figure 2D–G). The reflected values in Figure 2A–G correspond to the mean and standard deviation. 

LMWP, expressed as β-2-microglobulin excretion (Figure 2D), was 18-fold higher in the KI model compared to the WT group (*p* < 0.0001). Furthermore, β-2-microglobulin excretion in the KI model was doubled at day 31 after the beginning of the experiment (Supplementary Table S1). KI mice excreted 75% more calcium at T31 compared to the WT group (*p* < 0.05) (Supplementary Table S1), and the calcium excretion increased 8% as the experiment progressed (Figure 2E). At T21, we observed a significant difference in the phosphate excretion data in the KI group (*p* < 0.01) (Figure 2F), compared to the WT group, as is shown in Supplementary Table S1. This trend was maintained throughout the experiment, at the end of which, a 15% increase was observed, unlike in the WT group, in which the data remained stable. This significant difference reached its maximum at T31 (*p* < 0.05), when the KI mice excreted twice as much calcium as the WT group.

A progressive increase in urine output was observed as the experiment progressed, as shown in Figure 2G. At T31, a significant increase in the urinary excretion of the KI model was observed with respect to the WT group. In this case, the KI group excreted approximately 59% more than the WT group (*p* < 0.01). The results divided by interquartile range are shown in Supplementary Figure S2, where it is observed that KI mice present polyuria, LMWP, hypercalciuria and hyperphosphaturia, especially in medium ranges, compared to WT mice.

### 2.4. Effect of 4-PBA in the Phenotype of Knock-in Clcn5 Val523del Mice

**Figure 3:**
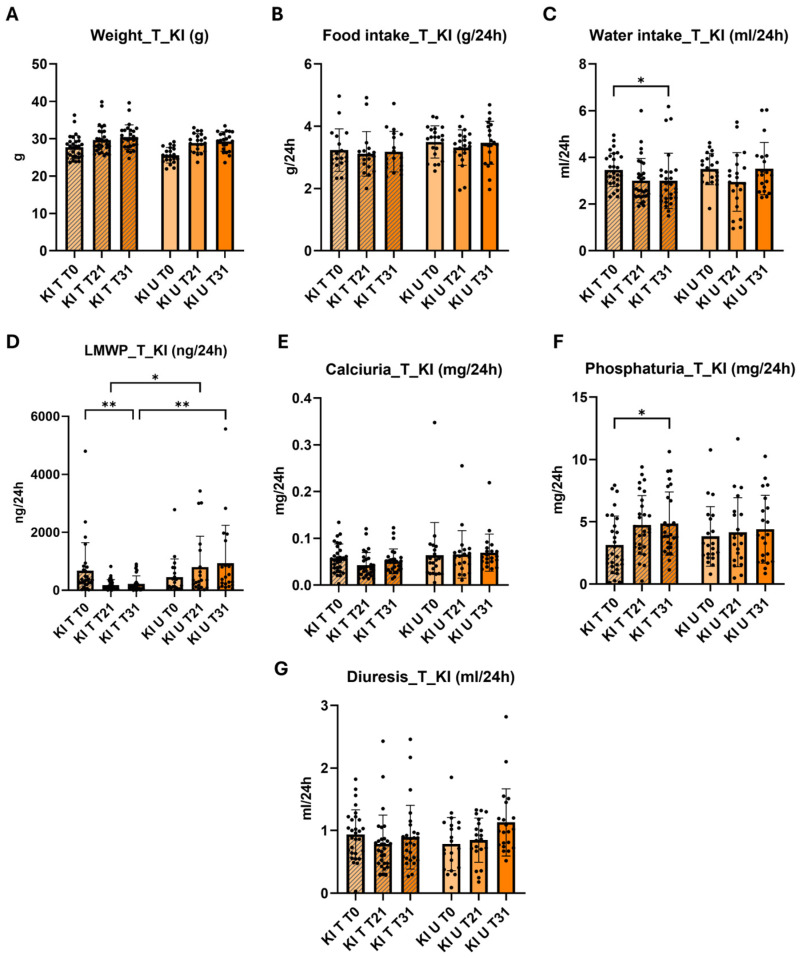
Results of 4-PBA treatment of the *Clcn5* Val523del mouse model. (**A**–**G**). Comparison of weight, food and water intake, LMWP, calciuria, phosphaturia and diuresis between KI treated (KI T) and untreated (KI U) mice. The bars correspond to the mean and standard deviations. Striped and unstriped bars correspond to KI T and KI U mice, respectively. * and ** correspond to *p* < 0.05 and *p* < 0.01 vs. Untreated/KI T T0 vs. KI T T31.

We then studied the effect of 4-PBA in β-2-microglobulin excretion, diuresis, calciuria, phosphaturia, weight, food intake and water intake of the KI mice. All mice selected for treatments and control experiments were males. As in the characterization results, the values represented in Figure 3 correspond to the mean and standard deviation of the results of the KI treated (KI T) and KI untreated (KI U) mice. (Figure 3) The results of the treated and untreated WT groups are shown in Supplementary Figure S3, where a significant increase in phosphaturia was found in the treated group (*p* < 0.01). No differences were observed in weight and food intake results in the treated (KI T) and untreated (KI U) groups (Figure 3A,B). A significant reduction in water intake was observed in KI T mice at T31 compared to KI T mice at T0 (*p* < 0.05) (Figure 3C).

The LMWP results showed a significant difference between the KI T and KI U groups at T21 (*p* < 0.01) and T31 (*p* < 0.001) (Figure 3D). At T21, β-2-microglobulin excretion decreased from 799.7 ng/24 h in the KI U group to 181 ng/24 in the treated mice, which represents a reduction of 77%. This reduction percentage was maintained at T31, where a decrease from 936.1 ng/24 h in the KI U group to 219.8 ng/24 h in the KI T group was observed (Figure 3D). The KI T group showed a significant reduction in β-2-microglobulin excretion at T21 (*p* < 0.001) and T31 (*p* < 0.001) (Figure 3D). At the end of the experiment, we observed a decrease from 675.8 ng/24 h at T0 to 219.8 ng/24 h at T31, which represents a 67% reduction. Regarding the WT mice, at T31, the excretion of the KI T group was six times higher than the WT T group, which contrasts with the U groups, where the difference was almost 20 times higher (Supplementary Tables S1 and S2). We performed a classification of the individuals of both KI groups based on their level of LMWP, and we observed that as the experiment progressed, in the case of the KI U group the number of mice with medium and high excretion values increased from 76% at T0 to 95% at T31. In the KI T group, the percentage of mice with medium and high LMWP excretion obtained at T0, as in the U group, was 75%, however, at T31, the percentage of mice with medium to high excretion values was 24%, which is a 71% reduction from the KI U group (Table 1).

Although there was no significant reduction in calcium excretion in KI T mice compared to KI U, a reduction in the variability of the data was observed (Figure 3E). Furthermore, when comparing the data from KI T mice with WT T mice, we observed that at T21 and T31, the significant difference in calcium excretion with respect to WT mice disappeared (Supplementary Table S2). Regarding the urinary phosphate levels observed in Figure 3F, the KI T group presented an increase in urinary phosphate excretion at T31 compared to T0 (*p* < 0.05). No differences were observed between the T and U groups at T31, both in the KI model and in the WT groups (Figure S2 and Figure 3F). No differences were observed in the diuresis of the KI T mice with respect to the KI U mice (Figure 3D), although we did find, as in the calcium excretion data, that the polyuria presented at T0 with respect to the WT T mice, disappeared at T21 and T31 (Supplementary Table S2). Supplementary Figure S4 shows the results of 4-PBA treatment in the KI model separated by interquartile range. We observed that the reduction in LMWP occurred at all levels. Calcium excretion was also significantly reduced at medium levels, whether we compare between T0 and T21 in the T group (*p* < 0.01) or when comparing between T and U groups at T21 (*p* < 0.001).

The analysis of the characterization data showed a significant difference between both groups (*p* = 0.017 *). The MANOVA analysis of the 4-PBA treatment results also showed a significant difference between the KI T and KI U groups (*p* = 0.01 *). This difference was higher in the β-2-microglobulin data, in which the *p*-value at T21 and T31 was 0.005 ** and 0.008 **, respectively. 

## 3. Discussion

DD-1 lacks therapeutic options beyond those aimed at palliating its symptoms, so it is important to develop a new strategy aimed at correcting or reducing the molecular effects caused by *CLCN5* mutations. As observed in the Western blot results presented in this manuscript and in the studies carried out by Durán et al. [28], the p.Val523del mutation affects the intracellular localization of the protein, but not its expression. The two existing KO mouse models for DD-1 do not express the ClC-5 protein [11,12]; therefore, they have not been used to test potential drug therapies. In the present study, we have generated a KI mouse of the C57BL/6 strain carrying *Clcn5* deletion p.Val523del, located in exon 10. This KI mouse expresses a mutated ClC-5 protein, so it could be useful for the study of molecular effects caused by the mutation and to test pharmacological treatments. We first verified that this mouse presented the phenotypic characteristics of DD-1. LMWP, which affects 100% of DD-1 cases, as well as hypercalciuria and hyperphosphaturia, present in 80% and 30% of patients, respectively, have been considered for this characterization [2,39]. Body weight, the amount of food and water ingested, and the amount of urine excreted in 24 h were also monitored.

LMWP was calculated by analyzing β-2-microglobulin in mice urine at 24 h. This low-molecular-weight protein is the most widely used for the calculation of LMWP in humans [40]. Throughout the experiment, an increase in the β-2-microglobulin excreted by the KI mice was observed, indicating the progression of the disease. At 12 weeks of age, 75% of the KI mice presented LMWP. DD-1 is a pediatric disease, so all mice are expected to have debuted with LMWP before adulthood. This lower percentage may be due to the fact that C57BL/6 mice are often resistant to developing proteinuria [41]. This variability could be a limitation for the applicability of our findings to other mouse models or to DD-1 patients. At 16 weeks of age, the percentage of mice with LMWP increased to 95%. Only one mouse presented a normal LMWP value, although in the upper range. On average, within 31 days, β-2-microglobulin excretion was twice as high as at the beginning of the experiment. Compared to WT mice, a 20-fold higher excretion of β-2-microglobulin was observed in the KI group at the end of the experiment. The great variability observed in the data, which was around 150

%, should be noted as a limitation of this study. However, several studies have shown that this variability also occurs in humans [40,42]. Likewise, the quantitative data of clara cell protein (CC16) excretion in the Guggino KO model also showed high variability [12]. Additionally, the total proteinuria calculated in mice of the C57BL/6 strain showed a deviation of practically 100% [43]. Stechman et al. attributed this variability in the murine models to random factors such as the social hierarchy, which can lead to aggressions and, therefore, a higher level of stress or the changes in the behavior and physiology of the animals when placed in the metabolic cages, even with a previous period of acclimatization, a factor that has already been reported in other studies [44,45]. Furthermore, other stress factors in the animals, such as their handling by the researcher, must also be considered [46,47,48]. The values of LMWP observed in our study are not comparable to others described in the literature, since in both the *Clcn5* KO models and in other models on the C57BL/6 strain, LMWP was measured using other low-molecular-weight proteins like CC16, non-quantitative measures of β-2-microglobulin, or just as total proteinuria [12,41,43].

The KI mouse group presented polyuria, with no significant differences in the amount of water ingested. As in the β-2-microglobulin data, polyuria increased as the experiment progressed, unlike in the WT group, in which the values remained stable. An upward progression was also observed in the calciuria and phosphaturia data of the KI group, in addition to a significant difference in both cases compared to the WT group. An increase in tubular PTH can induce an increase in 1-α-hydroxylase and therefore an elevation in calcitriol, as observed in KO models and in patients with DD-1 [11,12]. At the intestinal level, calcitriol would increase calcium absorption, so it would be expected that this increase would produce hypercalciuria and nephrolithiasis. However, a decrease in 25(OH)D endocytosis and a very significant increase in urine were also observed in KO mice, so it is believed that the balance between 25(OH)D and stimulation of 1-α-hydroxylase, both produced by a loss of ClC-5 function, would determine the presence or absence of hypercalciuria and that this balance could depend on genetic or nutritional factors [3,11,12,49,50]. The NHE3 exchanger also appears to play an important role in receptor-mediated endocytosis [51]. In this case, a reduction in its expression was also observed in both KO models and in PTH-induced endocytosis in the Jentsch model and could also explain the cases of metabolic alkalosis and hypokalemia in patients with DD-1 [3,52]. On the other hand, in the Jentsch KO model, hyperphosphaturia was associated with decreased NaPi2a expression. In KO models, PTH was found at normal levels in serum, but an increase of approximately 1.7-fold in urine was observed compared to WT mice, due to lack of internalization into the cell due to loss of PTH and megalin, which led to the conclusion that phosphaturia in KO mice could possibly be caused by decreased endocytosis of filtered PTH [3,11,12,13,49,52].

Compared to WT mice, our KI mice exhibited LMWP, hypercalciuria, hyperphosphaturia and polyuria. These results are comparable to the Guggino KO model [12], in which the same phenotypic characteristics were observed, and unlike the Jentsch model [11], which did not present hypercalciuria. These data confirm that our KI *Clcn5* Val523del mouse is a good model for the study of DD-1 since it presents the main clinical characteristics of the disease, especially LMWP. In addition, our model could be useful for the study of possible therapeutic options that help mitigate the disease symptoms. 

The deletion of valine 523 in ClC-5 causes, at least partially, an increase in stress in the ER due to the partial retention and accumulation of the mutated protein [28]. This mutation can also alter the endocytosis pathway and the ion transporter function of the protein. A broader focus on these pathways could provide a more comprehensive understanding of DD-1 pathology and treatment responses. Chemical chaperones are molecules capable of stabilizing a target protein, allowing it to exit the ER and thus reducing reticular stress. In this study, the therapeutic effect of one of these chaperones, 4-PBA, was evaluated in the KI *Clcn5* Val523del model. The drug, 4-PBA, has been approved by the European and American drug agencies for its current use for the treatment of metabolic diseases related to the urea cycle, since it reduces ammonia and glutamine concentrations in plasma [53]. On the other hand, it has also been observed in some studies that 4-PBA helps stabilize protein folding [31,33,38]. Its main mechanism of action is through the interaction of its hydrophobic regions with the exposed hydrophobic segments of misfolded proteins, preventing protein aggregation, promoting protein folding and preventing ER stress [54]. Evidence shows that 4-PBA modulates the misfolded protein response and acts as a histone deacetylase inhibitor, modulating chromatin remodeling and transcription by increasing histone acetylation [55] and regulating the expression of anti-apoptotic genes [56]. Its therapeutic potential has been widely established as a potential anti-cancer agent and in diseases caused by misfolded proteins, such as cystic fibrosis, thalassemia, spinal muscular atrophy, type 2 diabetes mellitus, amyotrophic lateral sclerosis, Huntington’s disease, Alzheimer’s disease and Parkinson’s disease [54,57,58,59,60,61,62]. In the kidney, it has been shown to attenuate ER stress and decrease apoptosis in renal tubule cells and renal interstitial fibrosis [35,36] and to reduce the concentration of albuminuria in mice with acute kidney injury [33]. Therefore, we hypothesized that 4-PBA could be useful to reduce LMWP in DD-1 patients with mutations that cause retention of the ClC-5 protein in the ER.

Drug repositioning offers several advantages over the search for new formulas to treat a disease. One of the biggest problems with the discovery of new molecules is the lack of security it offers [63]. According to data from Orphanet, only 10% of trials with new molecules give a positive therapeutic effect on the disease, so the development of new drugs aimed at treating rare diseases, due to their low prevalence, represents a very high economic investment. In this case, 4-PBA has already shown its safety in preclinical and human trials. In addition, the time required for its use as a possible treatment for DD-1 would be significantly reduced and the large financial investment required by new drugs both in their development stage and in phases I and II of tests would not be necessary in humans [63].

We observed a reduction of 77% in the excretion of β-2-microglobulin in the KI T group, which was maintained until the end of the experiment. In addition, the data variability was reduced in these mice, going from 150% in the untreated group to 100% in the treated group. On the other hand, a division by degree of LMWP was performed using the interquartile range, in which the same behavior was observed in all groups, so there was no difference in the action of 4-PBA depending on the degree of LMWP. Comparing the KI and the WT group, LMWP is still observed in the KI T mice, but this difference goes from being twenty times higher in the untreated group to six times higher in the treated group. As a percentage, 24% of the KI T mice exhibited medium or high levels of β-2-microglobulin excretion at T31, in contrast to 95% observed in the untreated group. This reduction in urinary β-2-microglobulin may be due to two factors: the 4-PBA may be helping the correct folding of ClC-5 and promoting its arrival at its destination, or as seen in previous studies, treatment with 4-PBA produces a cellular increase in megalin and cubilin levels, both in kidney lysate and in the plasma membrane [33]. It is possible to think that this increase in megalin and cubilin may be due to the chaperone effect, although Mohammed-Ali et al. also found not only an increase in these receptors in the cell membrane, but also an increase in mRNA levels, so further studies would be necessary in this regard [33]. The increase in megalin and cubilin could replace the loss of these proteins occurring in DD-1, due to the lack of recycling of the endosomes towards the membrane caused by the malfunction of ClC-5 [9,11,12,13]. In turn, this would produce an increase in the absorption of low-molecular-weight proteins, so the treatment with 4-PBA could be useful for different cases of DD-1, not just those in which the protein is retained in ER.

Regarding urine excretion, no significant differences were observed between the KI T and KI U groups, although the diuresis data of the KI T mice does not show the tendency to increase over time that is seen in the KI U group. KI T data remained stable throughout the experiment. Furthermore, in the comparison of the KI T mice with the WT T group, we observed that the KI mice presented polyuria at the beginning of the experiment and this significant difference was lost as the treatment with 4-PBA progressed. The results of calcium excretion were similar to those of diuresis. No significant differences were observed between the KI T and KI U groups, although the data did stabilize and the tendency to increase over time presented by the KI U mice was lost. We have no suitable explanation for this but perhaps it has to do with the fact that hypercalciuria is not a constant feature of DD-1. In addition, although it was not significant, we observed a decrease of approximately 30% in the data at T21 that was maintained at T31. It is also worth highlighting the decrease in the dispersion of the data of the KI T group. On the other hand, as in the diuresis data, when comparing the KI T group with the WT T at T0, the KI mice presented a significant increase in calcium excretion with respect to WT. This increase disappeared with treatment at T21 and T31. Also, the two treated groups, KI and WT, started with lower phosphaturia values than those of the untreated groups. In samples T21 and T31, an increase in excretion was observed, in which the values were equal to the untreated group. To date, no other study has described the effect of 4-PBA on these ions in urine.

In summary, our KI mouse model shows the main characteristics of DD-1 and is therefore a suitable model for testing therapeutic drugs. KI mice treated with 4-PBA exhibit significantly decreased β-2-microglobulin excretion. Further work will be needed to determine whether the treatment with 4-PBA reduces ER retention of the mutated ClC-5 protein in the kidney of KI mice and to establish whether the reduction in LMWP is directly related to a more efficient endocytosis process. Although interspecies variations in drug metabolism should be considered and clinical trials should be conducted to confirm the efficacy of 4-PBA in DD-1 patients, our results suggest that 4-PBA could be useful in slowing the progression of DD-1 in patients with *CLCN5* variant p.Val523del. Furthermore, we propose that this chaperone could be suitable for DD-1 patients with other mutations that result in partial or total retention of the ClC-5 protein in the ER.

## 4. Materials and Methods

### 4.1. Preparation of the Targeting Vector and Generation of Clcn5 Val523del Knock-in Mice

C57BL/6 mice carrying the c.1566_1568delTGT; p.Val523del mutation in the *Clcn5* gene were generated by PolyGene Transgenetics (Rümlang, Switzerland) using gene targeting in ES cells. The targeting vector contained two *Clcn5* homologous regions with the mutation in exon 10 and an FTR-flanked neomycin resistance cassette (Neo) inserted in intron 9 (Figure 1A). For the construction of this vector, the *Clcn*5 homology regions were amplified by PCR from a BAC DNA template (RP23-440N3), and the mutation was introduced by overlap extension PCR. The construct was verified by DNA sequencing. The targeting vector was then linearized with *Xho*I and electroporated into C57BL/6 ES cells. G418 was used to select for stable transfection. PCR, Southern blotting and sequencing were used to screen and validate clones. Positive ES-cell clones were injected into C57BL/6 ES blastocysts to generate chimeric mice. Lastly, the FTR-flanked neomycin cassette was excised by Flt recombination upon breeding with germ-line Flp-expressing mice. For this work, the principle of the three Rs was followed. Males and females were separated from each litter without there being more than six animals per cage at the time of carrying out the experiment. All mice were housed in ventilated cages in rooms with controlled temperature, humidity and a 12 h light/12 h dark cycle. Water and food were supplied ad libitum. These processes were performed in the laboratory animal facility of the University of La Laguna. The study protocol and animal procedures were approved by the Comité de Ética y Bienestar Animal (CEIBA) of University of La Laguna and the Dirección General De Ganadería de la Consejería de Agricultura, Ganadería, Pesca y Aguas del Gobierno de Canarias.

### 4.2. Genotyping

Genotyping of mice was performed between 4 and 6 weeks of age by standard polymerase chain reaction (PCR) procedures on DNA derived from ear tacking. Both DNA extraction and amplification were performed using the AccuStart™ II Mouse Genotyping Kit (Quanta Biosciences, Beverly, MA, USA) following the manufacturer’s instructions and the following PCR primers: E10genotF: 5′-ACTGACTCCCATGCTTTGCT-3′ and E10genotR: 5′-CATCACATCCATTGCCAGAG-3′. The obtained amplicons were purified with the NucleoSpin™ Gel and PCR Clean-up Kit (Macherey Nagel, Dueren, Germany) and sent to Macrogen Spain for sequencing. Sequences were compared in BLAST with the reference sequence for *Clcn5* (AF134117.1).

### 4.3. Western Blot

The expression of the murine ClC-5 protein in the kidney was analyzed by Western blot to confirm its expression in the *Clcn5* Val523del KI model. Total proteins were obtained from kidney lysates from WT and KI mice and separated by 10% sodium dodecyl sulphate polyacrylamide gel electrophoresis (SDS-PAGE). Proteins were transferred to a 0.45 µm nitrocellulose membrane (Bio-Rad, Hercules, CA, USA). The membrane was blocked with 5% nonfat milk powder in TBS buffer supplemented with 0.1% Tween 20 (Sigma-Aldrich, St. Louis, MO, USA) for 1 h at room temperature under shaking. To detect the protein, a ClC-5 Polyclonal Antibody (ThermoFisher Scientific, 1:1000, Waltham, MA, USA) was used. Actin was taken as a constitutive WT protein and was detected using the β-Actin Mouse mAB antibody (Cell Signaling Technology, 1:1000, Danvers, MA, USA). The membrane was analyzed in a Fusion Solo S chemiluminescence chamber (Vilber Lourmat, Collégien, France) using EvolutionCapt software v18.02 (Vilber Lourmat, Eberhardzell, Germany). Quantification of the bands was performed with the Fiji software (https://imagej.net/ accessed on 3 November 2023) [64] using the ROI Manager tool. The ratios represented are between the WT mouse and the KI for ClC-5, and for actin. The ratio between ClC-5 and actin was also obtained in both cases. The data obtained are the average of the quantification of two independent Western blots carried out. A *t*-test was performed to confirm whether significant differences existed.

### 4.4. Phenotypic Characterization

All mice selected for phenotypic characterization were males. At three months of age (T0), urine excreted in 24 h by 13 WT and 21 *Clcn5* Val523del KI mice was collected using metabolic cages with 24 h of prior acclimatization, in addition to the amount of water and food ingested in 24 h, individually. This collection was repeated 21 (T21) and 31 (T31) days later. Measurements related to the main phenotypic characteristics of DD-1 were performed on urine samples; β-2-microglobulin excretion using the Mouse Beta-2-Microglobulin ELISA Kit (Abcam, Cambridge, UK), calcium and phosphate, using the Calcium Colorimetric Assay Kit (Sigma-Aldrich, St. Louis, MO, USA) and Phosphate Assay Kit (Sigma-Aldrich, St. Louis, MO, USA), respectively, and creatinine in serum and urine, using the Mouse Creatinine ELISA Kit (My BioSource, San Diego, CA, USA). All assays were performed following the manufacturers’ instructions.

### 4.5. Treatment with 4-PBA

All mice selected for treatments and control experiments were males. Treatment with 4-PBA (Santa Cruz Biotechnology, Paso Robles, CA, USA) was administered in water for 31 days to 20 WT and 29 *Clcn5* Val523del KI mice randomly assigned to four groups: WT and KI mice receiving treatment (treatment group) and WT and KI mice receiving only water (WT group). Due to the variability in the doses used in previous studies, an intermediate dose of 250 mg/kg/day was established [65,66]. Serum (24 h) and urine samples were taken to re-determine the values of the mentioned characteristic clinical parameters of DD-1.

### 4.6. Statistical Analysis

Due to the nature of this experiment, we sought to obtain sufficient statistical power of the data to ensure the results. The sample size was calculated using the G*Power application v 3.1.9.7 (Heinrich-Heine-Universität, Düsseldorf, Germany) [67] (https://www.psychologie.hhu.de/arbeitsgruppen/allgemeine-psychologie-und-arbeitspsychologie/gpower accessed on 3 November 2023). Due to the lack of data from previous studies in this regard, both the mean and the variability of the groups were fixed with respect to the results obtained in the analysis of a group of five mice from each group (WT and KI). Statistical analysis of the results was performed with the GraphPad Prism 9 software v9.0.0 (GraphPad Software, San Diego, CA, USA) (https://www.graphpad.com/ accessed on 3 November 2023). For all data, normality tests were performed. Subsequently, and based on the results of the normality test, parametric (one-way ANOVA) or non-parametric tests (Kruskal–Wallis) were performed to compare groups. In addition, a multivariate analysis of variance (MANOVA) was performed using SPSS software v29.0 (IBM, Armonk, NY, USA) to analyze the difference between groups and the effect of 4-PBA on them. The variables β-2-microglobulin, phosphaturia and calciuria were included in this analysis. On the other hand, the β-2-microglobulin data from treated and untreated KI mice were divided into low, medium or high excretion groups established by the 25th and 75th percentiles. The statistical power of this analysis was 0.93.

## Figures and Tables

**Table 1:** Percentage of KI treated and untreated mice classified based on their β-2-microglobulin excretion. The percentages at T0, T21 and T31 are shown. This classification was made by analysis of quartiles, with low excretion being below the first quartile, average excretion between the second and third quartiles, and high excretion above the last quartile.

	T0			T21			T31		
	Low	Medium	High	Low	Medium	High	Low	Medium	High
Untreated	23.8%	52.4%	23.8%	14.3%	57.1%	28.6%	4.8%	57.1%	38.1%
Treated	24.1%	51.7%	24.1%	68.9%	27.6%	3.4%	75.9%	13.8%	10.3%

## Data Availability

The raw data supporting the conclusions of this article will be made available by the authors on request.

## Supplementary Materials

The following supporting information can be downloaded at: https://www.mdpi.com/article/10.3390/ijms25158110/s1.

## References

[1] Lieske J.C., Milliner D.S., Beara-Lasic L., Harris P., Cogal A., Abrash E., Adam M.P., Feldman J., Mirzaa G.M., Pagon R.A., Wallace S.E., Bean L.J.H., Gripp K.W., Amemiya A. (2012). Dent Disease. GeneReviews® [Internet].

[2] Claverie-Martín F., Ramos-Trujillo E., García-Nieto V. (2010). Dent’s disease: Clinical features and molecular basis. Pediatr. Nephrol..

[3] Anglani F., Gianesello L., Beara-Lasic L., Lieske J. (2019). Dent disease: A window into calcium and phosphate transport. J. Cell. Mol. Med..

[4] Gianesello L., Ceol M., Bertoldi L., Terrin L., Priante G., Murer L., Peruzzi L., Giordano M., Paglialonga F., Cantaluppi V. (2020). Genetic Analyses in Dent Disease and Characterization of *CLCN5* Mutations in Kidney Biopsies. Int. J. Mol. Sci..

[5] D’Ambrosio V., Wan E.R., Siew K., Hayes W., Walsh S.B. (2024). A female patient with Dent disease due to skewed X-chromosome inactivation. Clin. Kidney J..

[6] Lloyd S.E., Pearce S.H., Günther W., Kawaguchi H., Igarashi T., Jentsch T.J., Thakker R.V. (1997). Idiopathic low molecular weight proteinuria associated with hypercalciuric nephrocalcinosis in Japanese children is due to mutations of the renal chloride channel (*CLCN5*). J. Clin. Investig..

[7] Devuyst O. (2010). Dent’s disease: Chloride-proton exchange controls proximal tubule endocytosis. Nephrol. Dial. Transplant..

[8] Scheel O., Zdebik A.A., Lourdel S., Jentsch T.J. (2005). Voltage-dependent electrogenic chloride/proton exchange by endosomal CLC proteins. Nature.

[9] Novarino G., Weinert S., Rickheit G., Jentsch T.J. (2010). Endosomal Chloride-Proton Exchange Rather Than Chloride Conductance Is Crucial for Renal Endocytosis. Science.

[10] Bignon Y., Alekov A., Frachon N., Lahuna O., Doh-Egueli C.J.-B., Deschênes G., Vargas-Poussou R., Lourdel S. (2018). A novel *CLCN5* pathogenic mutation supports Dent disease with normal endosomal acidification. Hum. Mutat..

[11] Piwon N., Günther W., Schwake M., Bösl M.R., Jentsch T.J. (2000). ClC-5 Cl--channel disruption impairs endocytosis in a mouse model for Dent’s disease. Nature.

[12] Wang S.S., Devuyst O., Courtoy P.J., Wang X.-T., Wang H., Wang Y., Thakker R.V., Guggino S., Guggino W.B. (2000). Mice lacking renal chloride channel, CLC-5, are a model for Dent’s disease, a nephrolithiasis disorder associated with defective receptor-mediated endocytosis. Hum. Mol. Genet..

[13] Devuyst O., Jouret F., Auzanneau C., Courtoy P.J. (2005). Chloride Channels and Endocytosis: New Insights from Dent’s Disease and ClC-5 Knockout Mice. Nephron Physiol..

[14] Günther W., Lüchow A., Cluzeaud F., Vandewalle A., Jentsch T.J. (1998). ClC-5, the chloride channel mutated in Dent’s disease, colocalizes with the proton pump in endocytotically active kidney cells. Proc. Natl. Acad. Sci. USA.

[15] Moulin P., Igarashi T., Van Der Smissen P., Cosyns J.-P., Verroust P., Thakker R.V., Scheinman S.J., Courtoy P.J., Devuyst O. (2003). Altered polarity and expression of Hþ-ATPase without ultrastructural changes in kidneys of Dent’s disease patients. Kidney Int..

[16] Chang M.-H., Brown M.R., Liu Y., Gainullin V.G., Harris P.C., Romero M.F., Lieske J.C. (2020). Cl− and H+ coupling properties and subcellular localizations of wildtype and disease-associated variants of the voltage-gated Cl−/H+ exchanger ClC-5. J. Biol. Chem..

[17] Jentsch T.J., Pusch M. (2018). CLC chloride channels and transporters: Structure, function, physiology, and disease. Physiol. Rev..

[18] Shipman K.E., Weisz O.A. (2020). Making a Dent in Dent Disease. Function.

[19] Wright J., Morales M.M., Sousa-Menzes J., Ornellas D., Sipes J., Cui Y., Cui I., Hulamm P., Cebotaru V., Cebotaru L. (2008). Transcriptional adaptation to *Clcn5* knockout in proximal tubules of mouse kidney. Physiol. Genom..

[20] Yadav M.K., Yoo K.W., Atala A., Lu B. (2022). Lentiviral vector mediated gene therapy for type I Dent disease ameliorates Dent disease-like phenotypes for three months in ClC-5 null mice. Mol. Ther. Methods Clin. Dev..

[21] Wu F., Reed A.A., Williams S.E., Loh N.Y., Lippiat J.D., Christie P.T., Large O., Bettinelli A., Dillon M.J., Goldraich N.P. (2009). Mutational Analysis of CLC-5, Cofilin and CLC-4 in Patients with Dent’s Disease. Nephron Physiol..

[22] Ramos-Trujillo E., Claverie-Martin F., Garcia-Nieto V., Ariceta G., Vara J., Gonzalez-Acosta H., Garcia-Ramirez M., Fons J., Cordoba-Lanus E., Gonzalez-Paredes J. (2013). Dent’s disease: Identification of seven new pathogenic mutations in the *CLCN5* gene. J. Pediatr. Genet..

[23] Sekine T., Komoda F., Miura K., Takita J., Shimadzu M., Matsuyama T., Ashida A., Igarashi T. (2013). Japanese Dent disease has a wider clinical spectrum than Dent disease in Europe/USA: Genetic and clinical studies of 86 unrelated patients with low-molecular-weight proteinuria. Nephrol. Dial. Transplant..

[24] Park E., Choi H.J., Lee J.M., Ahn Y.H., Kang H.G., Choi Y.M., Park S.J., Cho H.Y., Park Y.-H., Lee S.J. (2014). Muscle involvement in Dent disease 2. Pediatr. Nephrol..

[25] Hureaux M., Ashton E., Dahan K., Houillier P., Blanchard A., Cormier C., Koumakis E., Iancu D., Belge H., Hilbert P. (2019). High-throughput sequencing contributes to the diagnosis of tubulopathies and familial hypercalcemia hypocalciuria in adults. Kidney Int..

[26] Dutzler R., Campbell E.B., Cadene M., Chait B.T., MacKinnon R. (2002). X-ray structure of a ClC chloride channel at 3.0 A reveals the molecular basis of anion selectivity. Nature.

[27] Wu F., Roche P., Christie P.T., Loh N.Y., Reed A.A., Esnouf R.M., Thakker R.V. (2003). Modeling study of human renal chloride channel (hCLC-5) mutations suggests a structural-functional relationship. Kidney Int..

[28] Durán M., Burballa C., Cantero-Recasens G., Butnaru C.M., Malhotra V., Ariceta G., Sarró E., Meseguer A. (2021). Novel Dent disease 1 cellular models reveal biological processes underlying ClC-5 loss-of-function. Hum. Mol. Genet..

[29] Tanaka K., Nakaki T. (2004). Reduced renal ClC-5 Cl− channel expression in spontaneously hypertensive rats with microalbuminuria. Eur. J. Pharmacol..

[30] Kim Y.E., Hipp M.S., Bracher A., Hayer-Hartl M., Ulrich Hartl F. (2013). Molecular Chaperone Functions in Protein Folding and Proteostasis. Annu. Rev. Biochem..

[31] Carlisle R.E., Brimble E., Werner K.E., Cruz G.L., Ask K., Ingram A.J., Dickhout J.G. (2014). 4-Phenylbutyrate Inhibits Tunicamycin-Induced Acute Kidney Injury via CHOP/GADD153 Repression. PLoS ONE.

[32] Wang Y.-J., Di X.-J., Mu T.-W. (2014). Using pharmacological chaperones to restore proteostasis. Pharmacol. Res..

[33] Mohammed-Ali Z., Lu C., Marway M.K., Carlisle R.E., Ask K., Lukic D., Krepinsky J.C., Dickhout J.G. (2017). Endoplasmic reticulum stress inhibition attenuates hypertensive chronic kidney disease through reduction in proteinuria. Sci. Rep..

[34] Yue J., Sun X., Duan X., Sun C., Chen H., Sun H., Zhang L. (2023). Triphenyl phosphate proved more potent than its metabolite diphenyl phosphate in inducing hepatic insulin resistance through endoplasmic reticulum stress. Environ. Int..

[35] Chiang C.-K., Hsu S.-P., Wu C.-T., Huang J.-W., Cheng H.-T., Chang Y.-W., Hung K.-Y., Wu K.-D., Liu S.-H. (2011). Endoplasmic Reticulum Stress Implicated in the Development of Renal Fibrosis. Mol. Med..

[36] Liu D., Ke Z., Luo J. (2016). Thiamine Deficiency and Neurodegeneration: The Interplay Among Oxidative Stress, Endoplasmic Reticulum Stress, and Autophagy. Mol. Neurobiol..

[37] Li Q., Zhang K., Hou L., Liao J., Zhang H., Han Q., Guo J., Li Y., Hu L., Pan J. (2023). Endoplasmic reticulum stress contributes to pyroptosis through NF-κB/NLRP3 pathway in diabetic nephropathy. Life Sci..

[38] Ni Y.-H., Deng H.-F., Zhou L., Huang C.-S., Wang N.-N., Yue L.-X., Li G.-F., Yu H.-J., Zhou W., Gao Y. (2022). Ginsenoside Rb1 Ameliorated Bavachin-Induced Renal Fibrosis via Suppressing Bip/eIF2α/CHOP Signaling-Mediated EMT. Front. Pharmacol..

[39] Gianesello L., Del Prete D., Ceol M., Priante G., Calò L.A., Anglani F. (2020). From protein uptake to Dent disease: An overview of the *CLCN5* gene. Gene.

[40] van Berkel Y., Ludwig M., van Wijk J.A.E., Bökenkamp A. (2016). Proteinuria in Dent disease: A review of the literature. Pediatr. Nephrol..

[41] Ishola D.A., van der Giezen D.M., Hahnel B., Goldschmeding R., Kriz W., Koomans H.A., Joles J.A., Hegarty J., Chiu D.Y.Y., Middleton R.J. (2005). In mice, proteinuria and renal inflammatory responses to albumin overload are strain-dependent. Nephrol. Dial. Transplant..

[42] Mansour-Hendili L., Blanchard A., Le Pottier N., Roncelin I., Lourdel S., Treard C., González W., Vergara-Jaque A., Morin G., Colin E. (2015). Mutation Update of the *CLCN5* Gene Responsible for Dent Disease 1. Hum. Mutat..

[43] Stechman M.J., Ahmad B.N., Loh N.Y., Reed A.A.C., Stewart M., Wells S., Hough T., Bentley L., Cox R.D., Brown S.D.M. (2010). Establishing normal plasma and 24-hour urinary biochemistry ranges in C3H, BALB/c and C57BL/6J mice following acclimatization in metabolic cages. Lab. Anim..

[44] Sharp J., Zammit T., Azar T., Lawson D. (2003). Stress-like responses to common procedures in individually and group-housed female rats. J. Am. Assoc. Lab. Anim. Sci..

[45] Späni D., Arras M., König B., Rülicke T. (2003). Higher heart rate of laboratory mice housed individually vs in pairs. Lab. Anim..

[46] Champy M., Selloum M., Piard L., Zeitler V., Caradec C., Chambon P., Auwerx J. (2004). Mouse functional genomics requires standardization of mouse handling and housing conditions. Mamm. Genome.

[47] Van Loo P.L.P., Van de Weerd H.A., Van Zutphen L.F.M., Baumans V. (2004). Preference for social contact versus environmental enrichment in male laboratory mice. Lab. Anim..

[48] Van Loo P.L.P., Van der Meer E., Kruitwagen C.L.J.J., Koolhaas J.M., Van Zutphen L.F.M., Baumans V. (2004). Long-term effects of husbandry procedures on stress-related parameters in male mice of two strains. Lab. Anim..

[49] Silva I.V., Blaisdell C.J., Guggino S.E., Guggino W.B., Devuyst O., Pham P.-T., Matsumoto N., Shih R.N.G., Jo O.D., Yanagawa N. (2000). PTH regulates expression of ClC-5 chloride channel in the kidney. Am. J. Physiol. Physiol..

[50] Maritzen T., Rickheit G., Schmitt A., Jentsch T. (2006). Kidney-specific upregulation of vitamin D3 target genes in ClC-5 KO mice. Kidney Int..

[51] Gekle M., Völker K., Mildenberger S., Freudinger R., Shull G.E., Wiemann M. (2004). NHE3 Na+/H+ exchanger supports proximal tubular protein reabsorption in vivo. Am. J. Physiol. Physiol..

[52] Günther W., Piwon N., Jentsch T.J. (2003). The ClC-5 chloride channel knock-out mouse–an animal model for Dent’s disease. Pflügers Arch..

[53] Carretero-Colomer M. (2004). Fenilbutirato de sodio. Offarm.

[54] Iannitti T., Palmieri B. (2011). Clinical and Experimental Applications of Sodium Phenylbutyrate. Drugs R D.

[55] Perlmutter D.H. (2002). Chemical chaperones: A pharmacological strategy for disorders of protein folding and trafficking. Pediatr. Res..

[56] Ryu H., Smith K., Camelo S.I., Carreras I., Lee J., Iglesias A.H., Dangond F., Cormier K.A., Cudkowicz M.E., Brown R.H. (2005). Sodium phenylbutyrate prolongs survival and regulates expression of antiapoptotic genes in transgenic amyotrophic lateral sclerosis mice. J. Neurochem..

[57] Singh O.V., Vij N., Mogayzel P.J., Jozwik C., Pollard H.B., Zeitlin P.L. (2006). Pharmacoproteomics of 4-phenylbutyrate-treated IB3-1 cystic fibrosis bronchial epithelial cells. J. Proteome Res..

[58] Villani S., Dematteis G., Tapella L., Gagliardi M., Lim D., Corazzari M., Aprile S., Del Grosso E. (2023). Quantification of the Chemical Chaperone 4-Phenylbutyric Acid (4-PBA) in Cell Culture Media via LC-HRMS: Applications in Fields of Neurodegeneration and Cancer. Pharmaceuticals.

[59] Özcan U., Yilmaz E., Özcan L., Furuhashi M., Vaillancourt E., Smith R.O., Görgün C.Z., Hotamisligil G.S. (2006). Chemical Chaperones Reduce ER Stress and Restore Glucose Homeostasis in a Mouse Model of Type 2 Diabetes. Science.

[60] Qi X., Hosoi T., Okuma Y., Kaneko M., Nomura Y. (2004). Sodium 4-Phenylbutyrate Protects Against Cerebral Ischemic Injury. Mol. Pharmacol..

[61] Ricobaraza A., Cuadrado-Tejedor M., Marco S., Pérez-Otaño I., García-Osta A. (2010). Phenylbutyrate Rescues Dendritic Spine Loss Associated with Memory Deficits in a Mouse Model of Alzheimer Disease. Hippocampus.

[62] Zeng M., Sang W., Chen S., Chen R., Zhang H., Xue F., Li Z., Liu Y., Gong Y., Zhang H. (2017). 4-PBA inhibits LPS-induced inflammation through regulating ER stress and autophagy in acute lung injury models. Toxicol. Lett..

[63] Pushpakom S., Iorio F., Eyers P.A., Escott K.J., Hopper S., Wells A., Doig A., Guilliams T., Latimer J., McNamee C. (2019). Drug repurposing: Progress, challenges and recommendations. Nat. Rev. Drug Discov..

[64] Schindelin J., Arganda-Carreras I., Frise E., Kaynig V., Longair M., Pietzsch T., Preibisch S., Rueden C., Saalfeld S., Schmid B. (2012). Fiji: An open-source platform for biological-image analysis. Nat. Methods.

[65] Butchbach M.E., Lumpkin C.J., Harris A.W., Saieva L., Edwards J.D., Workman E., Simard L.R., Pellizzoni L., Burghes A.H. (2016). Protective effects of butyrate-based compounds on a mouse model for spinal muscular atrophy. Exp. Neurol..

[66] Hayashi G., Labelle-Dumais C., Gould D.B. (2018). Use of sodium 4-phenylbutyrate to define therapeutic parameters for reducing intracerebral hemorrhage and myopathy in Col4a1 mutant mice. Dis. Model. Mech..

[67] Faul F., Erdfelder E., Buchner A., Lang A.-G. (2009). Statistical power analyses using G*Power 3.1: Tests for correlation and regression analyses. Behav. Res. Methods.

