# Supplementary material for: 4-Phenylbutyric Acid Treatment Reduces Low-Molecular-Weight Proteinuria in a Clcn5 Knock-in Mouse Model for Dent Disease-1
Source: Int J Mol Sci. 2024 Jul 25;25(15):8110. doi: 10.3390/ijms25158110 (PMC11311629; doi:10.3390/ijms25158110)
Supplement: Supplementary file 1 [file ijms-25-08110-s001.zip › ijms-3091880-supplementary.pdf]

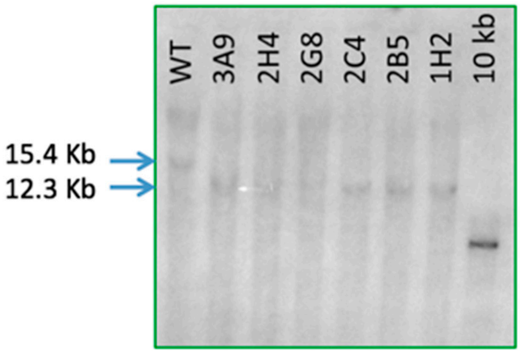

Supplementary Figure S1. Confirmation of homologous recombination by Southern blotting. Genomic DNA derived from positive embryonic stem (ES) cell clones was digested with restriction enzyme *Sph*I. A 3’ external probe was used for detection of the *Clcn5* allele. The analysis confirmed correct homologous recombination in all clones tested; the 15.4-kb band is from the wild-type (WT) genomic DNA present in the control ES cells, and the 12.3-kb band is from the correctly targeted allele.

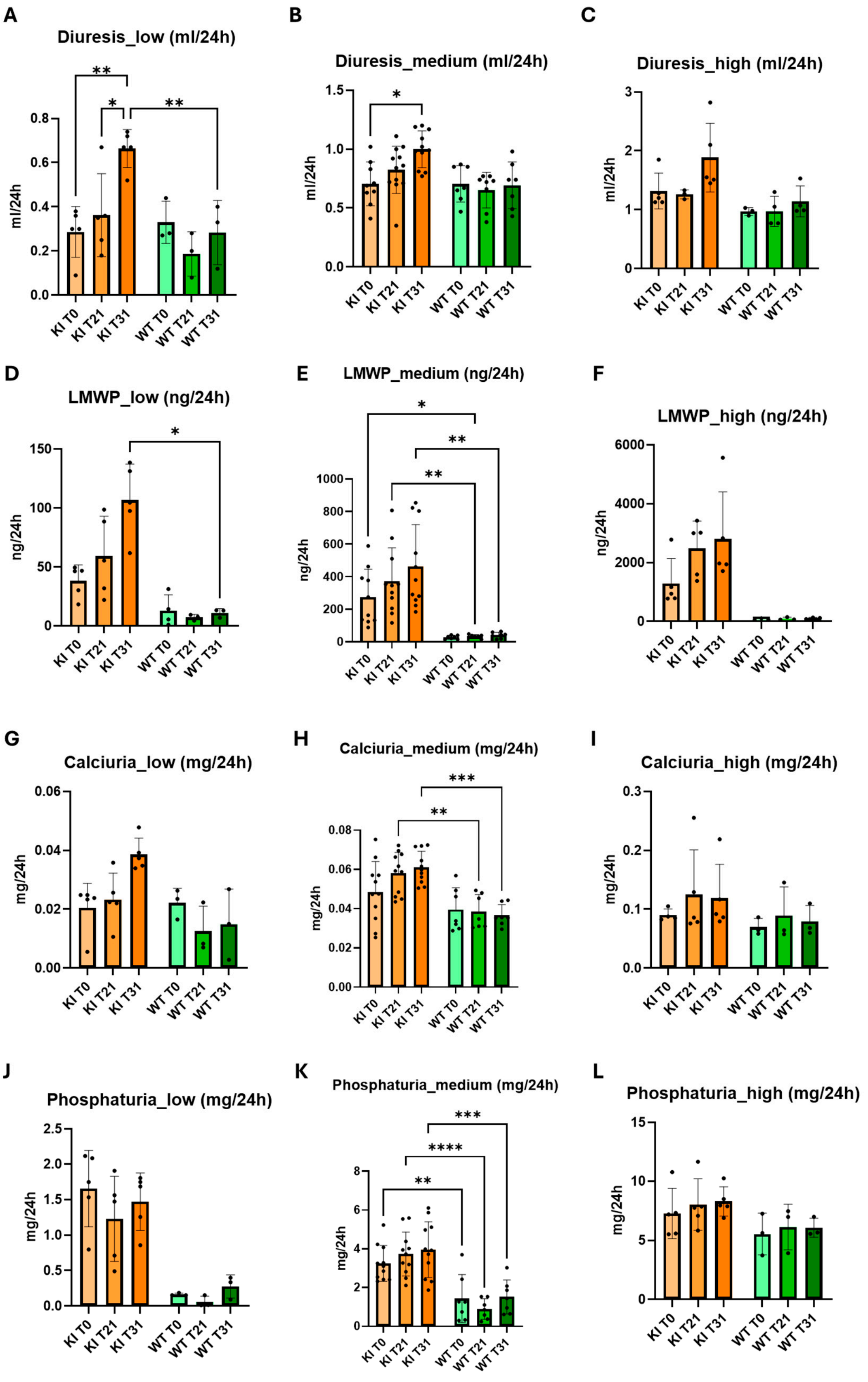

*, **, ***, ****: <0,05, <0,01, <0,001, <0,0001 KI vs. WT

Supplementary Figure S2. Results of clinical indicators of DD-1 in the characterization of the KI model separated by interquartile ranges. A-L**.** Comparison between KI and WT of LMWP, diuresis, calciuria and phosphaturia. The bars correspond to the mean and standard deviations. The data were separated according to the interquartile range: low (<25%), medium (25-75%), high (>75%).

	Weight (g)	*Water intake (ml/24h)*	*Food intake (g/24h)*	*Diuresis (ml/24h)*	*LMWP (ng/24h)*	*Calciuria (mg/24h)*	*Phosphaturia (mg/24h)*
WT T0	25.3 (±3.6)	2.6 (±0.6)	3.6 (±0.7)	0.68 (±0.26)	33.74 (±28.86)	0.04 (±0.02)	2.1 (±2.3)
KI T0	25.6(±2.1)	3.5 (±0.7)*	3.5 (±0.5)	0.78 (±0.42)	450.6 (±631.9)***	0.06 (±0.07)	3.8 (±2.4)
WT T21	27.9 (±3.0)	2.9 (±0.8)	3.1 (±0.7)	0.63 (±0.34)	41.04 (±38.26)	0.04 (±0.03)	1.9 (±2.6)
KI T21	28.9 (±2.5)	3.0 (±1.3)	3.3 (±0.6)	0.84 (±0.35)	799.7 (±1069)****	0.06 (±0.05)	4.2 (±2.7)**
WT T31	27.8 (±2.2)	3.1 (±0.9)	3.5 (±0.7)	0.71 (±0.38)	49.62 (±40.66)	0.04 (±0.03)	2.3 (±2.3)
KI T31	29.3 (±2.6)	3.5 (±1.1)	3.5 (±0.7)	1.13 (±0.54)*	936.1 (±1309)****	0.07 (±0.04)*	4.4 (±2.7)*

Supplementary Table S1. Numerical results of the characterization data shown in Figure 3. The means and standard deviations of the results obtained are shown. *. **.***. ****: P<0.05. <0.01. <0.001. <0.0001 vs. Control (WT).

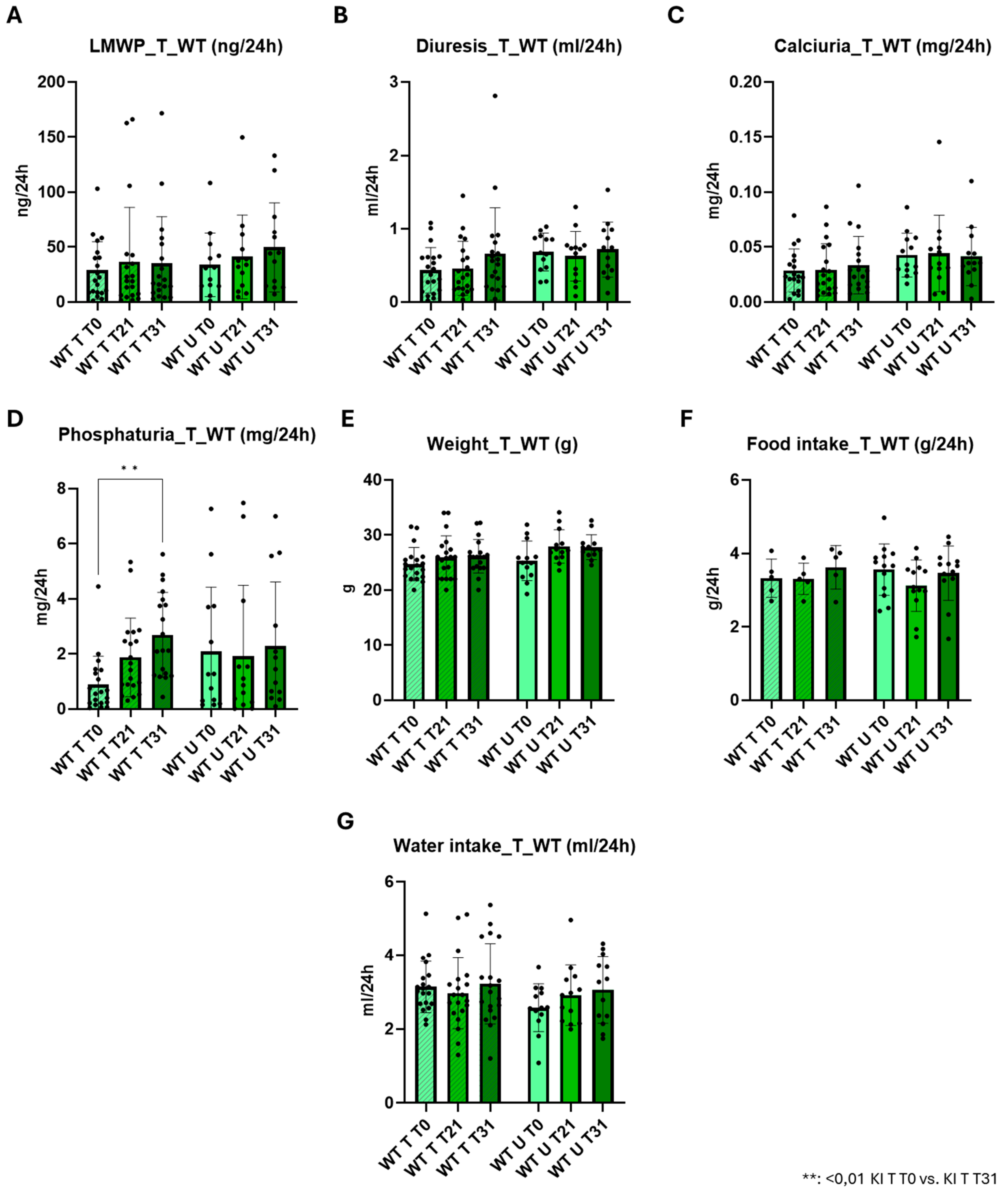

Supplementary Figure S3. Results of 4-PBA treatment in the *Clcn5 Val523del* mouse model. A-G. Comparison between control treated (WT T) and untreated (WT U) of LMWP, diuresis, calciuria, phosphaturia, weight, food and water intake. The bars correspond to the mean and standard deviations. Striped and unstriped bars correspond to WT T and WT U mice, respectively.

	Weight (g)	***Water intake (ml/24h)***	***Food intake (g/24h)***	***Diuresis (ml/24h)***	***LMWP (ng/24h)***	***Calciuria (mg/24h)***	***Phosphaturia (mg/24h)***
WT T T0	24.7 (±3.0)	3.1 (±0.7)	3.3 (±0.5)	0.43 (±0.31)	28.66 (±26.15)	0.03 (±0.02)	0.9 (±1.0)
KI T T0	27.7 (±3.1)*	3.5 (±0.7)	3.2 (±0.7)	0.94 (±0.39)***	675.8 (±965.1)****	0.06 (±0.03)**	3.1 (±2.3)**
WT T T21	25.9 (±3.9)	3.0 (±1.0)	3.3 (±0.4)	0.46 (±0.37)	36.4 (±49.72)	0.03 (±0.02)	1.9 (±1.4)
KI T T21	29.6 (±3.6)**	3.0 (±0.9)	3.1 (±0.7)	0.78 (±0.46)	181.0 (±186.6)**	0.04 (±0.03)	4.7 (±2.3)***
WT T T31	26.1 (±3.1)	3.2 (±1.1)	3.6 (±0.6)	0.66 (±0.63)	35.03 (±42.72)	0.03 (±0.03)	2.7 (±1.5)
KI T T31	30.4 (±3.3)***	3.0 (±1.2)	3.2 (±0.6)	0.89 (±0.51)	219.8 (±276.7)*	0.05 (±0.03)	4.9 (±2.5)

Supplementary Table S2. Numerical results of the 4-PBA treatment data shown in Figure 3 and Supplementary Figure 3. The means and standard deviations of the results obtained are shown. *. **. ***. ****: P<0.05. <0.01. <0.001. <0.0001 vs. WT T.

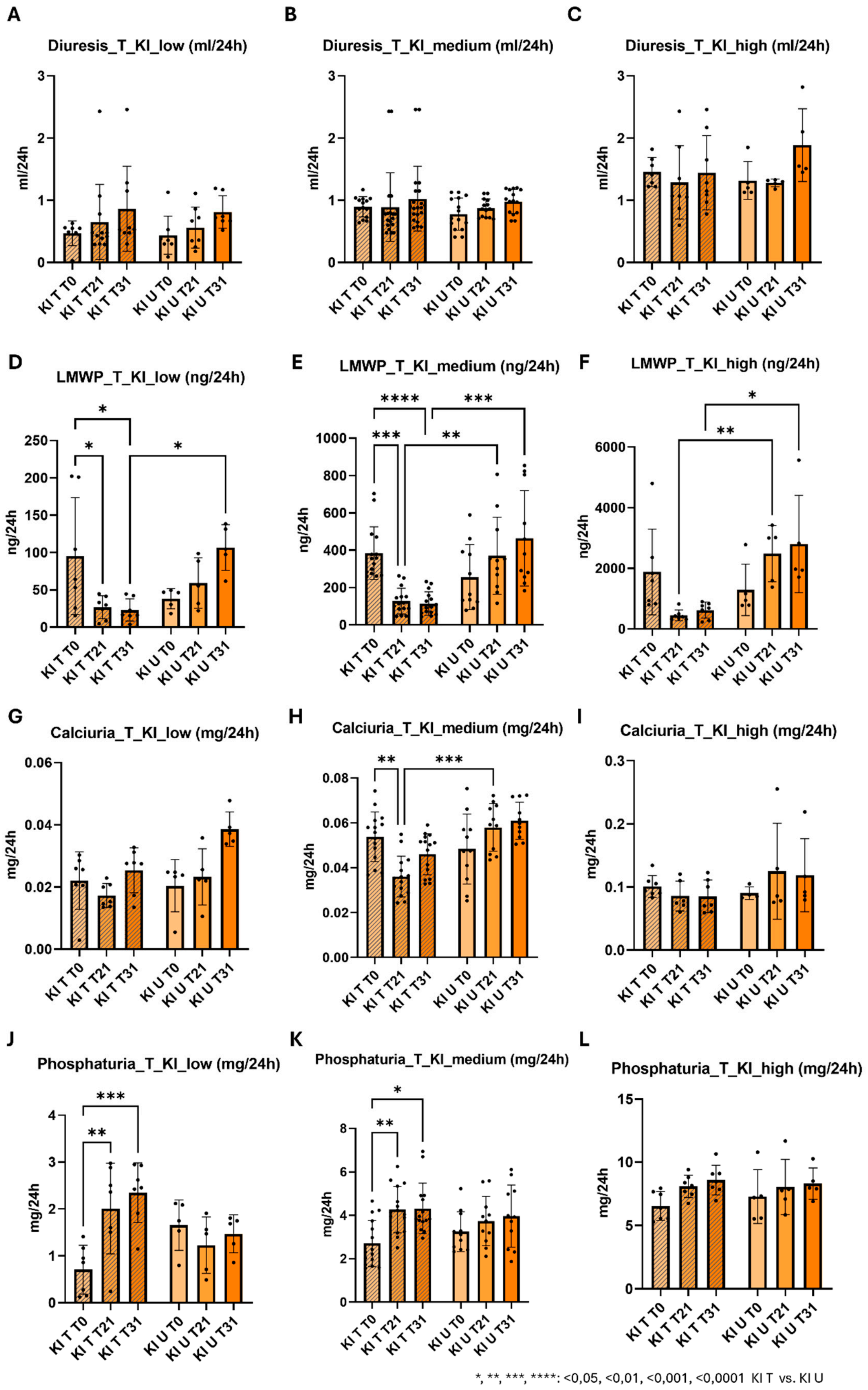

Supplementary Figure S4. Results of clinical indicators of DD-1 in 4-PBA treatment of KI model separated by interquartile ranges. A-L. Comparison between KI T and KI U of LMWP, diuresis, calciuria and phosphaturia. The bars correspond to the mean and standard deviations. The data were separated according to the interquartile range: low (<25%), medium (25-75%), high (>75%).
